# The mitochondria‒paraspeckle axis regulates the survival of transplanted stem cells under oxidative stress conditions

**DOI:** 10.7150/thno.88764

**Published:** 2024-01-27

**Authors:** Meng Zhao, Shuyun Liu, Yizhuo Wang, Ke Lv, Peng Lou, Pingya Zhou, Jiaying Zhu, Lan Li, Jingqiu Cheng, Yanrong Lu, Jingping Liu

**Affiliations:** 1 Department of General Surgery and NHC Key Laboratory of Transplant Engineering and Immunology, Frontiers Science Center for Disease-related Molecular Network, West China Hospital, Sichuan University, Chengdu 610041, China.; 2 Department of Emergency, Guizhou Provincial People's Hospital, Guiyang 550002, China.

**Keywords:** mesenchymal stem cells, oxidative stress, mitochondria, paraspeckle, cell death

## Abstract

**Rationale:** Stem cell-based therapies have emerged as promising tools for tissue engineering and regenerative medicine, but their therapeutic efficacy is largely limited by the oxidative stress-induced loss of transplanted cells at injured tissue sites. To address this issue, we aimed to explore the underlying mechanism and protective strategy of ROS-induced MSC loss.

**Methods:** Changes in TFAM (mitochondrial transcription factor A) signaling, mitochondrial function, DNA damage, apoptosis and senescence in MSCs under oxidative stress conditions were assessed using real-time PCR, western blotting and RNA sequencing, etc. The impact of TFAM or lncRNA nuclear paraspeckle assembly transcript 1 (NEAT1) knockdown or overexpression on mitochondrial function, DNA damage repair, apoptosis and senescence in MSCs was also analyzed. The effect of mitochondrion-targeted antioxidant (Mito-TEMPO) on the survival of transplanted MSCs was evaluated in a mouse model of renal ischemia/reperfusion (I/R) injury.

**Results:** Mitochondrial ROS (mtROS) bursts caused defects in TFAM signaling and overall mitochondrial function, which further impaired NEAT1 expression and its mediated paraspeckle formation and DNA repair pathways in MSCs, thereby jointly promoting MSC senescence and death under oxidative stress. In contrast, targeted inhibition of the mtROS bursts is a sufficient strategy for attenuating early transplanted MSC loss at injured tissue sites, and coadministration of Mito-TEMPO improved the local retention of transplanted MSCs and reduced oxidative injury in ischemic kidneys.

**Conclusions:** This study identified the critical role of the mitochondria‒paraspeckle axis in regulating cell survival and may provide insights into developing advanced stem cell therapies for tissue engineering and regenerative medicine.

## Introduction

Stem cell-based therapies have emerged as promising means for tissue engineering and regenerative medicine 1,2. For example, mesenchymal stem cell (MSC)-based therapies have shown substantial promise for the treatment of multiple ischemic diseases, such as ischemic heart and kidney injury 3. However, the outcomes of MSC therapies in different preclinical or clinical trials are controversial. One of the main reasons for this is insufficient cell survival or functional impairment of transplanted MSCs in harsh microenvironments (*e.g.*, persistent oxidative stress and inflammation) associated with disease 4,5. Notably, the fate (rapid loss or long-term survival) and outcomes of therapeutic MSCs are dependent on the tissue microenvironment in which they are transplanted. It has been shown that only a small number (~1.5%) of MSCs remain alive at 72 h after being locally transplanted into rat hind limb ischemic sites 6. Thus, it is important to elucidate the underlying mechanisms of early transplanted MSC loss in injured sites, as these findings will provide insights into advanced MSC-based therapies.

To date, there is an abundance of evidence indicating that oxidative stress (*e.g.,* ROS) is one of the primary factors that causes early MSC loss after transplantation 7,8. In general, moderate levels of ROS may function as signals to promote cell survival, whereas high levels of ROS can lead to cell death. Implanted MSCs generally undergo persistent ROS and proinflammatory substrate stimulation at injured tissue sites, which can disrupt the cellular redox balance and induce a senescent phenotype and/or cell death of MSCs 4,5,7. As a result, the overall survival and tissue repair potential of implanted MSCs are largely impaired 4,5. Although the possibility that ROS can induce MSC loss has been widely proposed, the underlying mechanisms remain elusive. Recently, mitochondria have been determined to be critical mediators of stem cell fate and function. Mitochondria are the center of cellular energy metabolism, and they also regulate many cellular events, such as cell proliferation, differentiation and cell death 9,10. Mitochondrial lesions, such as mitochondrial ROS (mtROS) bursts and mitochondrial DNA (mtDNA) defects, are strongly associated with the senescence and death of stem cells 9,10. We have found mitochondrial dysfunction (*e.g.,* reduced levels of TFAM, mtDNA copy number, and TOM20) and an increased senescence phenotype (*e.g.*, p53 and* β-gal*) in MSCs under oxidative stress conditions 11. Thus, the potential link between mitochondrial injury and MSC death under stress needs to be elucidated, as this may provide insights into strategies for improving transplanted MSC survival at injured sites.

**Figure 1:**
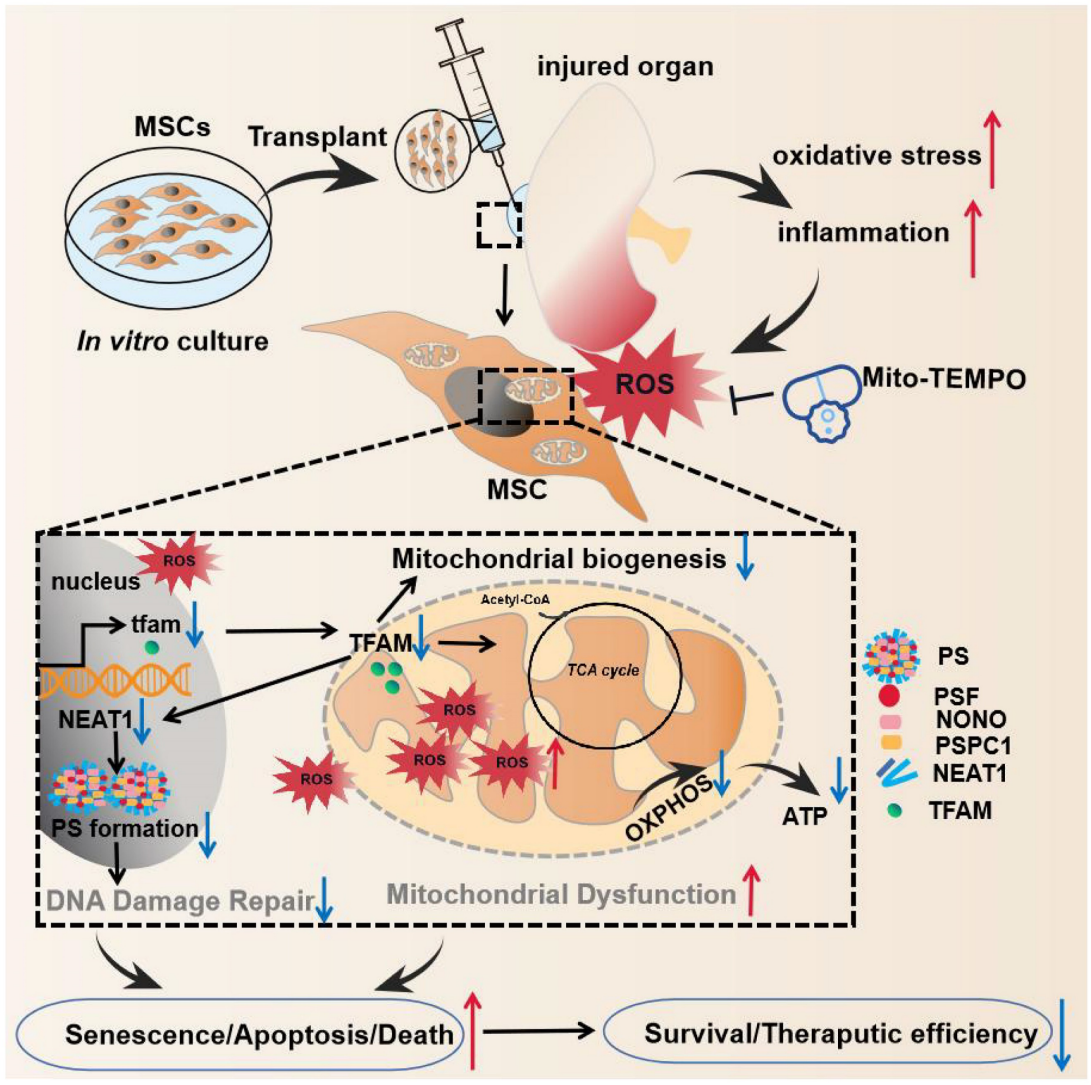
The key findings of this study. In the injured tissues, mtROS burst induced TFAM defects and mitochondrial injury of the transplanted MSCs, which further impaired NEAT1 expression and paraspeckle formation (disrupted mitochondria-paraspeckle axis), thereby promoting early loss of the transplanted MSCs. Targeted inhibition of the mtROS burst is a potent strategy for restoring TFAM signaling and reducing transplanted MSC loss in injured sites.

Here, we report that disruption of the mitochondria‒paraspeckle axis is a critical mechanism of underlying MSC death under oxidative stress, and can be attenuated through the use of a targeted mtROS inhibition strategy (Figure 1). Briefly, the mtROS burst induced TFAM signaling defects and severe mitochondrial injury in MSCs, which further impaired nuclear paraspeckle assembly transcript 1 (NEAT1) expression and its mediated paraspeckle formation and the DNA damage repair machinery, thereby promoting MSC senescence and death. Conversely, coadministration of a mitochondrion-targeted antioxidant (Mito-TEMPO) improved the survival of transplanted MSCs in ischemic sites. This study identifies the vital role of the mitochondria‒paraspeckle axis in regulating stem cell fate and provides insights into advanced stem cell therapies for regenerative medicine.

## Materials and Methods

### Cell culture and oxidative injury model

Human umbilical cord mesenchymal stem cells (MSCs) were obtained from the Sichuan Neo-life Stem Cell Biotech & Sichuan Stem Cell Bank (Chengdu, China), and their cell characterization and trilineage differentiation potential were confirmed by the company. MSCs were expanded under standard culture conditions (37 °C, 21% O_2_, 5% CO_2_) in Dulbecco's Modified Eagle Medium (DMEM, Gibco, Grand Island, NY, USA) supplemented with 10% fetal bovine serum (FBS, Gibco) and 1% penicillin and streptomycin (Beyotime Biotechnology, Shanghai, China). All the experiments were performed using cells at passages 3-5. To induce the oxidative injury model, MSCs were incubated with H_2_O_2_ (0.4 mM, Sigma‒Aldrich, USA) for 72 h.

### Mitochondrial morphology observation

MSCs were stained with 50 nM MitoTracker Green and Hoechst 33258 (Invitrogen, USA) at 37 °C for 30 min. After washing with PBS, representative images were observed via confocal microscopy (Nikon, N-STORM & A1, Tokyo, Japan). The aspect ratio (AR, major axis/minor axis) and form factor (FF, 4π × (area/perimeter^2^)) were calculated using ImageJ software (NIH) as previously reported 12.

### Staining for senescence-associated *β*-galactosidase (SA-*β*-gal)

The expression of senescence-associated-galactosidase (SA-*β*-gal) in MSCs was measured with a commercial staining kit (Beyotime Biotechnology, China) according to the manufacturer's instructions. The number of SA-*β*-gal-positive cells was counted using ImageJ software (NIH).

### Oxygen consumption rate (OCR) assay

The mitochondrial OCR of MSCs was measured on a Seahorse XF-24 Flux Analyzer (Seahorse Biosciences, Agilent, USA) using a Mitochondrial Stress Test (MST) assay kit as previously described 11. After treatment, MSCs (3 × 10^4^ per well) were plated in a XFe24-well culture microplate and adhered at 37 °C for 2 h with 5% CO_2_. Then, the growth medium was subsequently changed to assay medium, after which the cells were incubated for 60 min at 37 °C in a non-CO_2_ incubator. After that, the MST assay compounds were added to the probe plate following the protocol with oligomycin (1 μM), FCCP (2 μM), and rotenone/antimycin A (0.5 μM), and the OCR (pmol O_2_/min) of the cells was recorded.

### Cell apoptosis analysis

After treatment, the MSCs were collected and stained with an Annexin V-FITC/PI Apoptosis Detection Kit (BD, USA) according to the manufacturer's protocols. In brief, the cells were incubated with 5 μL of FITC-Annexin V and 1 μL of propidium iodide (PI) at room temperature for 15 min. After washing with PBS, the percentage of apoptotic MSCs was measured using flow cytometry (BD, USA).

### Western blotting

Cells were lysed in radioimmunoprecipitation assay (RIPA) buffer supplemented with protease inhibitors (Beyotime Biotechnology, China) and phosphatase inhibitors (CWBIO, Beijing, China) on ice. The protein concentration was determined by a BCA Protein Assay Kit (CWBIO, Beijing, China). The protein expression in the MSCs was assayed by sodium dodecyl sulfate‒polyacrylamide gel (SDS‒PAGE) electrophoresis after which the proteins were subsequently transferred to polyvinylidene difluoride (PVDF) membranes (Merck, Millipore). The membranes were blocked with 5% nonfat milk and incubated with primary antibodies against rabbit anti-TFAM (A1962, ABclonal), rabbit anti-TFAM (22586-1-AP, Proteintech), rabbit anti-p53 (10442-1-AP, Proteintech), rabbit anti-p21 (A19094, ABclonal), rabbit anti-BRCA1 (A11034, ABclonal), rabbit anti-COXIV (11242-1-AP, Proteintech), rabbit anti-NONO (A5282, ABclonal), rabbit anti-PSPC1 (A9209, ABclonal), rabbit anti-sirt1 (13161-1-AP, Proteintech), mouse anti-PSF (sc-271796, Santa Cruz), rabbit anti-phospho-histone H2A.X (#9718, 20E3, Cell Signaling Technology), rabbit anti-ATF2 (A2155, ABclonal), rabbit anti-phospho-BRCA1 (#9009, ser 1524, Cell Signaling Technology), rabbit anti-RPA32 (A2189, ABclonal) and rabbit anti-4HNE (ab46545, Abcam) and mouse anti-GAPDH (AC002, ABclonal) at 4 °C overnight. After washing with PBST, the PVDF membrane was incubated with horseradish peroxidase-conjugated secondary antibody (ZB2301, Zhongshanjinqiao Biotechnology) at 37 °C for 1 h. An enhanced chemiluminescence kit (Millipore) was used for signal detection. Protein bands were quantified using ImageJ software and normalized to the expression of GAPDH.

### Quantitative real-time PCR

Total RNA was extracted using TRIzol reagent (Life Technologies, USA) and reverse-transcribed into cDNA using a cDNA synthesis kit (Vazyme, China). Real-time polymerase chain reaction (Real-time PCR) was performed on a CFX96 real-time PCR detection system (Bio-Rad, USA) with SYBR Green (Vazyme, China). The primers used in this study are listed in Table S1. The data were analyzed using Bio-Rad CFX Manager software, and the relative change in mRNA expression was calculated by the delta-delta Ct method with GAPDH as an internal reference gene.

### Small interfering RNA (siRNA) and plasmid transfection

MSCs were transfected with *TFAM* siRNA (siTFAM*,* 100 pmol, GenePharma Biotechnology, China) or* NEAT1* siRNA (siNEAT1*,* 100 pmol, Tsingke Biotechnology Co., Ltd) using jetPRIME transfection reagent (Polyplus, Illkirch, France) for 5 h and then switched to fresh complete medium and cultured for an additional 24 h before experiments. MSCs transfected with scrambled siRNA were used as normal controls (NC MSCs). For overexpression of TFAM (TFAM-OE) or ATF2 (ATF2-OE) in MSCs, cells were transfected with a *TFAM* plasmid (Vigene Biosciences, Shandong, China) or ATF2 plasmid (SinoBiological, Beijing, China) using the jetPRIME transfection reagent for 5 h. Afterwards, the medium was changed to fresh complete medium and the cells were cultured for another 48 h.

### RNA sequencing (RNA-seq) assay of cells

Total RNA was extracted from MSCs treated with NC or siTFAM and exposed to H_2_O_2_ for different durations (0 h, 24 h, 48 h, 72 h) using TRIzol reagent (Life Technologies, USA), and genomic DNA was removed using DNase I (Takara, Shiga, Japan). RNA quality was determined using a 2100 Bioanalyzer (Agilent Technologies) and quantified using ND-2000 (NanoDrop Technologies). RNA-seq transcriptome libraries were constructed using a TruSeqTM RNA sample preparation kit (Illumina Inc., San Diego, CA, USA), and high-throughput sequencing was performed on an Illumina NovaSeq 6000 system at Shanghai Majorbio Bio-Pharm Technology Co., Ltd. (Shanghai, China). The expression of each transcript was calculated according to the fragments per kilobase of exon per million mapped reads (FRKM) method. Principal component analysis (PCA), volcano plots, and heatmaps were generated using an online platform (https://www.omicsolution.com/wkomics/main/).

### RNA in situ hybridization

The expression of lncRNA Neat1 in MSCs was detected using fluorescence in situ hybridization (FISH) as previously described 13. The transcribed antisense probes for Neat1 (Table S2) were synthesized by GenePharma (Shanghai, China) and labeled with Alexa Fluor 488 (Neat1_-_1) and Cy3 (Neat1_-_2). The cell slides were fixed with 4% paraformaldehyde in PBS for 15 min and then incubated with 0.1% Triton X-100 for 15 min. After washing with PBS, the slides were incubated with 2 × saline sodium citrate (SSC) at 37 °C for 30 min, followed by stepwise dehydration at 70%, and 85%, confluence and twice in 100% ethanol for 3 min. The slides were incubated with probes at 37 °C overnight. After that, the slides were washed with buffer solution, and nuclei were stained with DAPI (Sigma, USA). The stained slides were imaged by confocal laser scanning microscopy (Nikon, N-STORM & A1).

### Immunofluorescence (IF) staining

The cell slides or tissue sections were fixed with 4% paraformaldehyde in PBS for 10 min at room temperature and then permeabilized with 0.03% Triton X-100 (Sigma‒Aldrich, USA) for 10 min. After blocking in 1% BSA for 1 h, the slides were incubated with rabbit anti-phospho-histone H2A. X (#9718, 20E3, Cell Signaling Technology), rabbit anti-NONO (A5282, ABclonal), rabbit anti-PSPC1 (A9209, ABclonal), mouse anti-PSF (sc-271796, Santa Cruz), Goat anti-KIM-1 (AF1817, R&D Systems, Minneapolis, USA) and mouse anti-8-OHdG (sc-393871, Santa Cruz) antibodies overnight at 4 °C. After washing with PBS, the slides were incubated with the corresponding fluorescein isothiocyanate (FITC, Life Technologies)-conjugated or tetramethylrhodamine (TRITC, Abcam)-conjugated secondary antibody for 1 h at 37 °C. Nuclei were stained with DAPI (Sigma, USA) for 5 min and then washed with PBS. The stained slides were imaged by confocal laser scanning microscopy (Nikon, N-STORM & A1).

### Labeling of transplanted MSCs

To track the transplanted cells *in vivo*, MSCs were labeled with lipophilic near-infrared dyes (DID) as previously reported 14. Before transplantation, 5 µL of the DID dye solution (5 mg/mL, Invitrogen) was added to the MSC suspension (1 × 10^6^/mL), which was subsequently incubated for 15 min at 37 °C in the dark. To remove excessive dye, the stained cells were washed three times with PBS before use.

### Mouse model of ischemic kidney injury

All animal experiments were approved by the Ethics Committee of West China Hospital of Sichuan University (Permit number: 2020413A) and conducted according to the National Institutes of Health (NIH) guidelines. Male C57BL/6 mice (~20-25 g) were purchased from the Experimental Animal Center of Sichuan University (Chengdu, China). Animals were housed in individual cages with a standard environment, diet and water. The ischemic kidney injury model was generated as previously described 15. In brief, mice were anesthetized with 1% pentobarbital sodium (Merck) solution, and ischemic kidney injury was induced by bilateral clamping of the renal pedicles for 30 min. After all surgical procedures, the fascia and skin were closed in two layers.

### In vivo tracking of transplanted MSCs

MSCs were transplanted into injured mouse kidneys with or without Mito-TEMPO (MT) coadministration. In brief, mice with ischemic kidney injury were randomly divided into three groups (n = 6): control (PBS), MSCs, ATF2 OE-MSCs and MSCs + MT. After reperfusion of the kidney, DID-labeled MSCs (2 × 10^5^ cells in 50 μL PBS) were locally injected under the injured renal capsule using an insulin syringe, and the kidneys in the control group received 50 μL of PBS. For the MSC + MT groups, after MSC transplantation, the kidneys of the mice were immediately treated with MT solution (100 μL, 0.2 μM in DMSO/PBS, Santa Cruz). At the indicated time points, mice were sacrificed by an overdose of anesthesia, and their organs were collected and imaged on an optical imaging system (IVIS Spectrum, PerkinElmer, Waltham, MA, USA) to detect the surviving MSCs and the expression of 8-OHdG. The fluorescence signal of DID-labeled MSCs in each organ was normalized to that of the PBS group.

### Statistical analysis

All the data are presented as the mean ± SD. Student's t test or one-way ANOVA with Tukey's post hoc analysis was performed to compare the differences between two groups or among groups using GraphPad Prism 9.0 software (GraphPad Software Inc., La Jolla, CA), and p < 0.05 was considered a significant difference.

## Results and Discussion

### Oxidative stress induced TFAM defects and mitochondrial damage in MSCs

**Figure 2:**
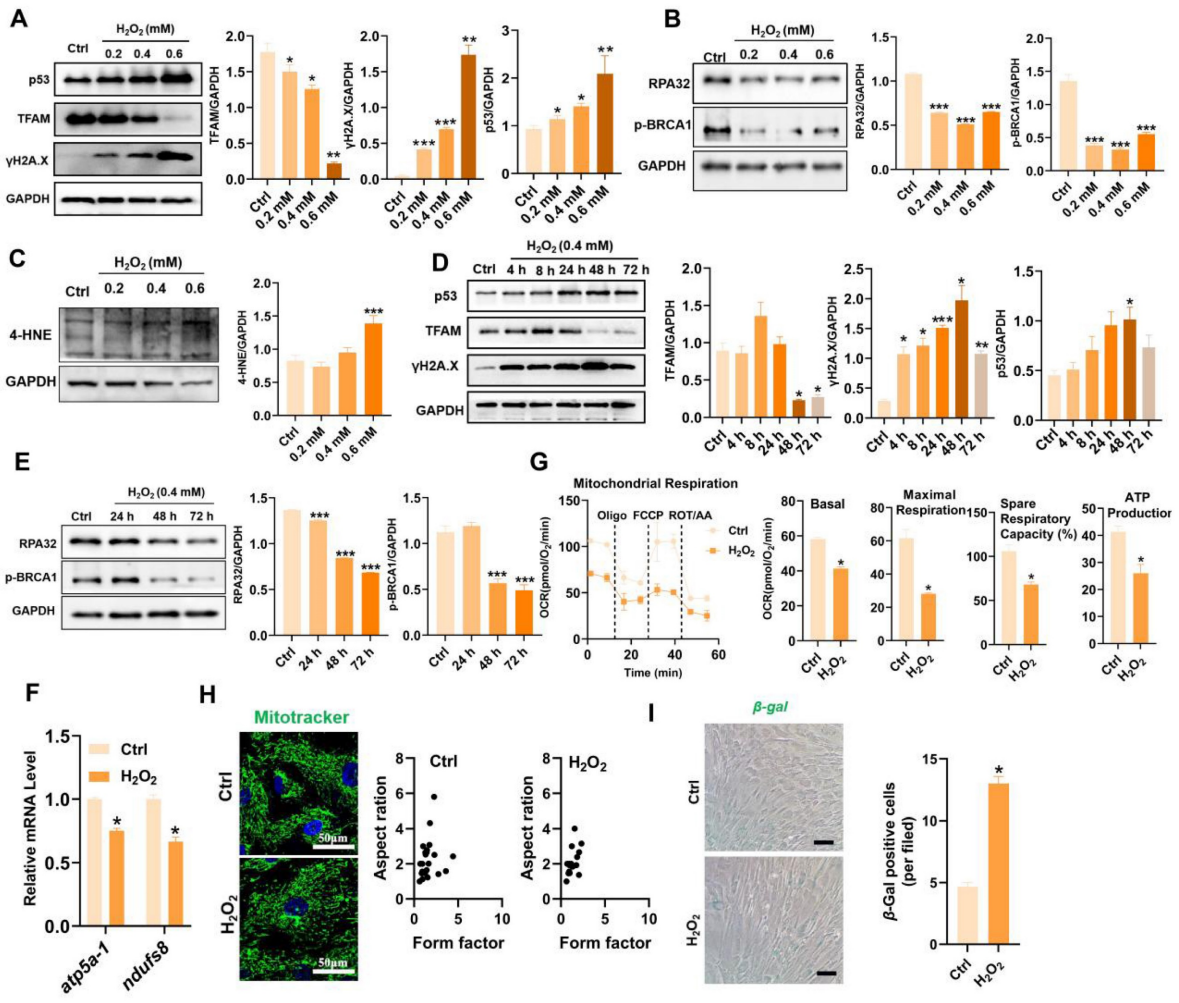
Oxidative stress caused TFAM defects and mitochondrial dysfunction in MSCs. (A-C) Western blot analysis and quantification of the protein levels of the TFAM, γ-H2A.X, p53, RPA32, p-BRCA1and 4-HNE in hMSCs treated with different concentrations of H_2_O_2_ (0 mM, 0.2 mM, 0.4 mM and 0.6 mM) for 72 h. (n = 3; *p < 0.05 *vs.* Ctrl group; **p < 0.01 *vs.* Ctrl group; ***p < 0.001 *vs.* Ctrl group). (D-E) Western blot analysis and quantification of the protein levels of the p53, TFAM, γ-H2A.X, RPA32 and p-BRCA1 proteins in hMSCs treated with 0.4 mM H_2_O_2_ for different durations (0 h, 4 h, 8 h, 24 h, 48 h and 72 h). (n = 3; *p < 0.05 *vs.* Ctrl group; **p < 0.01 *vs.* Ctrl group; ***p < 0.001 *vs.* Ctrl group). (F) Real-time PCR analysis of* atp5a-1* and *ndufs8* mRNA in hMSCs treated with 0.4 mM H_2_O_2_ for 72 h (n = 3; *p < 0.05 *vs.* Ctrl group). (G) Measurement of the mitochondrial oxygen consumption ratio (OCR) of hMSCs (n = 3; *p < 0.05 *vs.* Ctrl group). (H) Representative micrographs of Mito-Tracker Green staining in hMSCs (scale = 50 µm) and quantification of mitochondrial fragmentation. (I) *β*-gal staining and quantification were performed in hMSCs treated with or without H_2_O_2_ (0.4 mM for 72 h) (n = 3; *p < 0.05 *vs.* Ctrl group).

**Figure 3:**
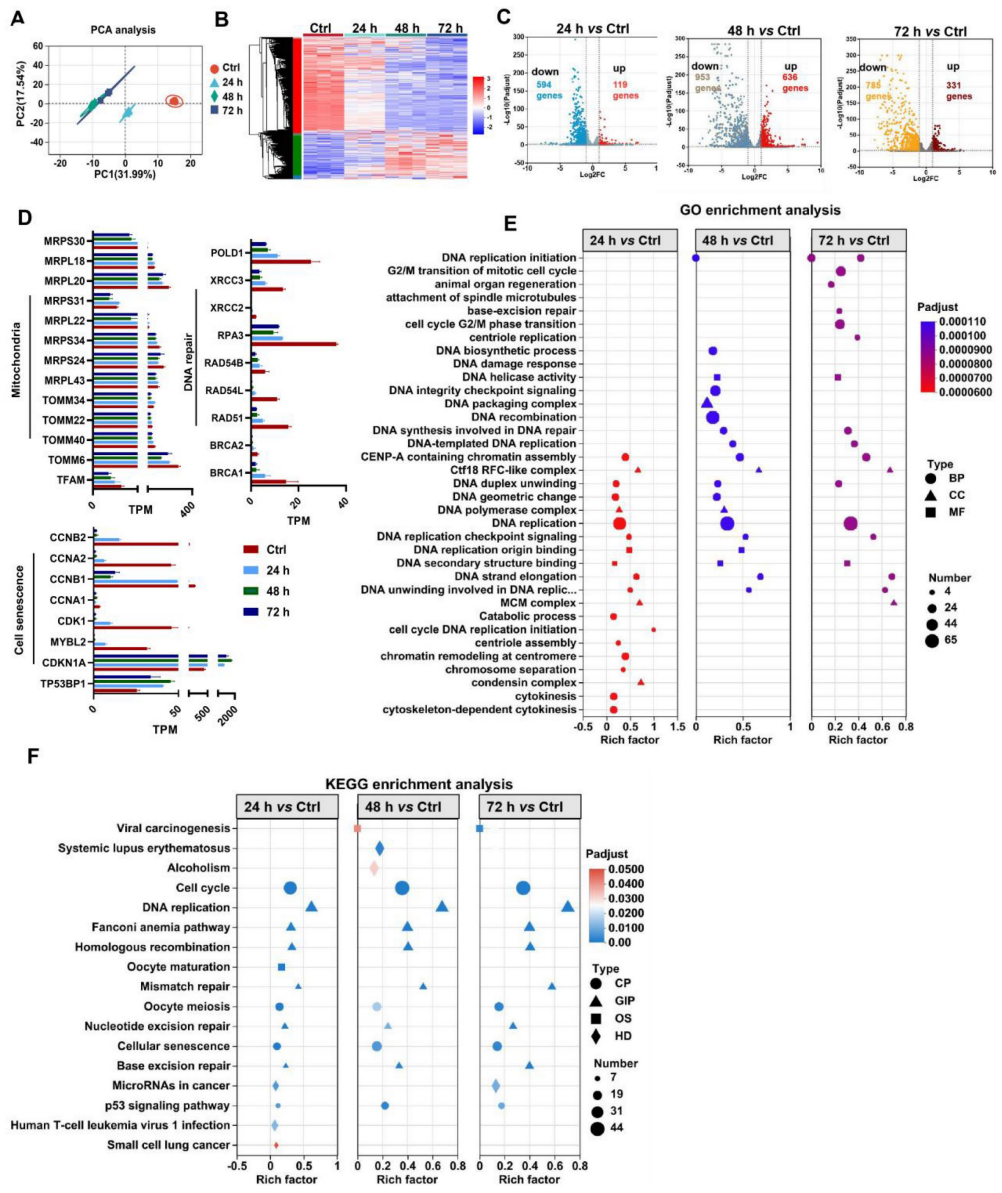
Oxidative stress causes mitochondrial dysfunction, DNA damage repair and senescence in MSCs. (A) PCA score plot representing discrepancies between groups (n = 3). (B) Heatmap showing the gene expression pattern of MSCs. (C) Volcano plot identified the differentially expressed genes (DEGs) between two groups (fold change >2, p < 0.05). (D) Quantitative analysis of DEGs between two groups. (E-F) Go and KEGG enrichment analysis showing cellular processes and pathways between groups.

MSC-based therapies are proposed to be potent means of promoting tissue repair in many types of organ injury 1,2. However, the survival and therapeutic efficacy of MSCs are largely impaired by the harsh microenvironment (*e.g.,* oxidative stress) at injured or diseased sites 7,8. Excessive production of reactive oxygen species (ROS) plays a critical role in the progression of tissue injury and can cause oxidative damage to diverse cellular contents, such as lipids, proteins, and nucleic acids 16. Notably, ROS can disrupt the cellular redox balance and induce the senescence phenotype and apoptosis in stem cells 17. In vivo stress conditions are complicated and multiple types of ROS, such as superoxide anion (O_2_·-), hydroxyl radical (OH·-) and hydrogen peroxide (H_2_O_2_), can be generated in the injured tissues. Moreover, fully mimicking such conditions in vitro is difficult. In many previous studies, H_2_O_2_ was used as one of the major ROS inducers for assessing the responses of cells to oxidative stress, since it is a key endogenous source of cellular ROS and can mimic the oxidative environment that may occur *in vivo* 18. According to previous reports, H_2_O_2_ concentrations ranging from 0.01 mM to 0.4 mM are frequently used as pathophysiologically relevant conditions to mimic oxidative stress-induced cell damage 19,20. H_2_O_2_ can arrest cell proliferation and induce a senescent phenotype (*e.g.,* p53/p21, SA-*β*-gal) in multiple types of cultured cells, including MSCs 21. Thus, we first assayed the impact of a ROS burst (induced by H_2_O_2_) on cell DNA damage and senescence in MSCs. H_2_O_2_ stimulation increased the degree of DNA damage (γ-H2A.X) and senescence (*β*-gal-positive cells and p53), as well as decreased DNA repair (*e.g.,* RPA32 and p-BRCA1) in MSCs in a time- and dose-dependent manner compared to those in control group (Figure 2A-B, D-E and I). These results confirm that oxidative stress is a vital trigger of transplanted MSC loss under tough conditions, which can induce severe DNA damage and senescence in MSCs. In particular, excessive ROS can also lead to oxidative damage to multiple biomolecules (*e.g.,* proteins, DNAs, lipids) directly or indirectly, which in turn leads to enhanced cell senescence 22,23. For example, increased levels of 4-hydroxy-2-nonenal (4-HNE, a marker of lipid peroxidation) were strongly associated with cell senescence 24. In this study, we found that H_2_O_2_-treated MSCs had increased levels of 4-HNE in a dose-dependent manner (Figure 2C). To explore the underlying mechanism of ROS-induced damage in MSCs, we performed RNA sequencing on MSCs exposed to 0.4 mM H_2_O_2_ for different durations (Figure 3). Principal component analysis (PCA) scatter plots and heatmaps showed an distinct differences in gene expression among the Ctrl, 24 h, 48 h, and 72 h groups (Figure 3A-B). The differentially expressed genes (DEGs) affected by ROS were identified using volcano plot (Figure 3C). For example, H_2_O_2_ treatment increased the expression of senescence-related genes, such as CDKN1A and TP53PB1, while it downregulated many DNA damage repair-associated genes, such as BRCA1, BRCA2, RPA3 and XRCC3, compared to those in the Ctrl group (Figure 3D). Further GO, KEGG and Reactome enrichment analyses indicated that these DEGs were enriched in multiple pathways, such as the cell cycle, signal transduction and DNA damage repair (Figure 3E-F and S3). Notably, disrupted expression of DNA repair genes, such as BRCA1, RAD51 and POLD1 was observed in MSCs after H_2_O_2_ exposure for 72 h (Figure 3D). Moreover, the expression of key mitochondria-related genes, such as TFAM and TOMM6, was also impaired in MSCs after long-term H_2_O_2_ treatment (Figure 3D). Overall, these results suggest that ROS can disrupt mitochondrial function and DNA damage repair in MSCs and thus promote cellular senescence. However, the specific mechanism by which ROS induce MSC senescence and death is not completely understood.

Mitochondria play central roles in regulating stem cell fate through the TCA cycle metabolism and ETC function 10, as they are the major sites of cellular energy (ATP production) and substrate metabolism, as well as intracellular ROS (~90%) production. Mitochondria are the major source and primary victim of ROS, and mtROS bursts play vital roles in the onset and progression of cell senescence and aging-related diseases 25. It has been reported that a mtROS burst can cause mtDNA damage and thus disrupt mitochondrial biogenesis and OXPHOS 12. Consistent with these findings, H_2_O_2_ stimulation suppressed the expression of TFAM (a key regulator of mitochondrial biogenesis) and ETC genes (*e.g., atp5a-1* and *ndufs8*) in MSCs (Figure 2A, D and F). Consequently, compared with those in the control group, the mitochondrial morphology in the H_2_O_2_-induced MSCs showed obvious fragmentation by Mito-Tracker Green staining (Figure 2H). In addition, MSCs exposed to H_2_O_2_ had decreased levels of mitochondrial mass (*e.g.,* TOM20, Figure S1A-B), mitochondrial related genes (*e.g.,* MRPS30, MRPS34 and MRPS31, Figure 3D), mitochondrial OCR and TCA cycle metabolites (*e.g.,* citrate, oxalacetate, fumarate and succinate) compared to those in the control group (Figure 3G, S2A-B). MRPS30, MRPS34 and MRPS31 are nuclear-encoded mitochondrial ribosomal proteins (MRPs) that are essential for the functional integrity of the mitoribosome complex as well as the biogenesis of the oxidative phosphorylation system 26. A previous study reported that mutations (or absence) in ribosome assembly proteins may be lethal or slow growth 27. Mitochondrial respiratory chain defects can further increase electron leakage and mtROS generation, resulting in a detrimental cycle that causes irreversible cell damage 28. As a result, MSCs in the H_2_O_2_ group exhibited increased levels of cell apoptosis and senescence compared to those in the control groups (Figure 2I and S1C). In addition to mitochondria, several other organelles, such as endoplasmic reticulum, can also be injured by oxidative stress 29. Overall, these results suggest that oxidative stress may cause damage to diverse types of organelles and biomolecules within cells and thus contribute to enhanced MSC senescence. Mitochondria are essential for maintaining MSC survival, and ROS-induced mitochondrial injury may be a critical reason for MSC loss (senescence and death) at injured sites.

### TFAM defects promoted MSC senescence/death under oxidative stress

**Figure 4:**
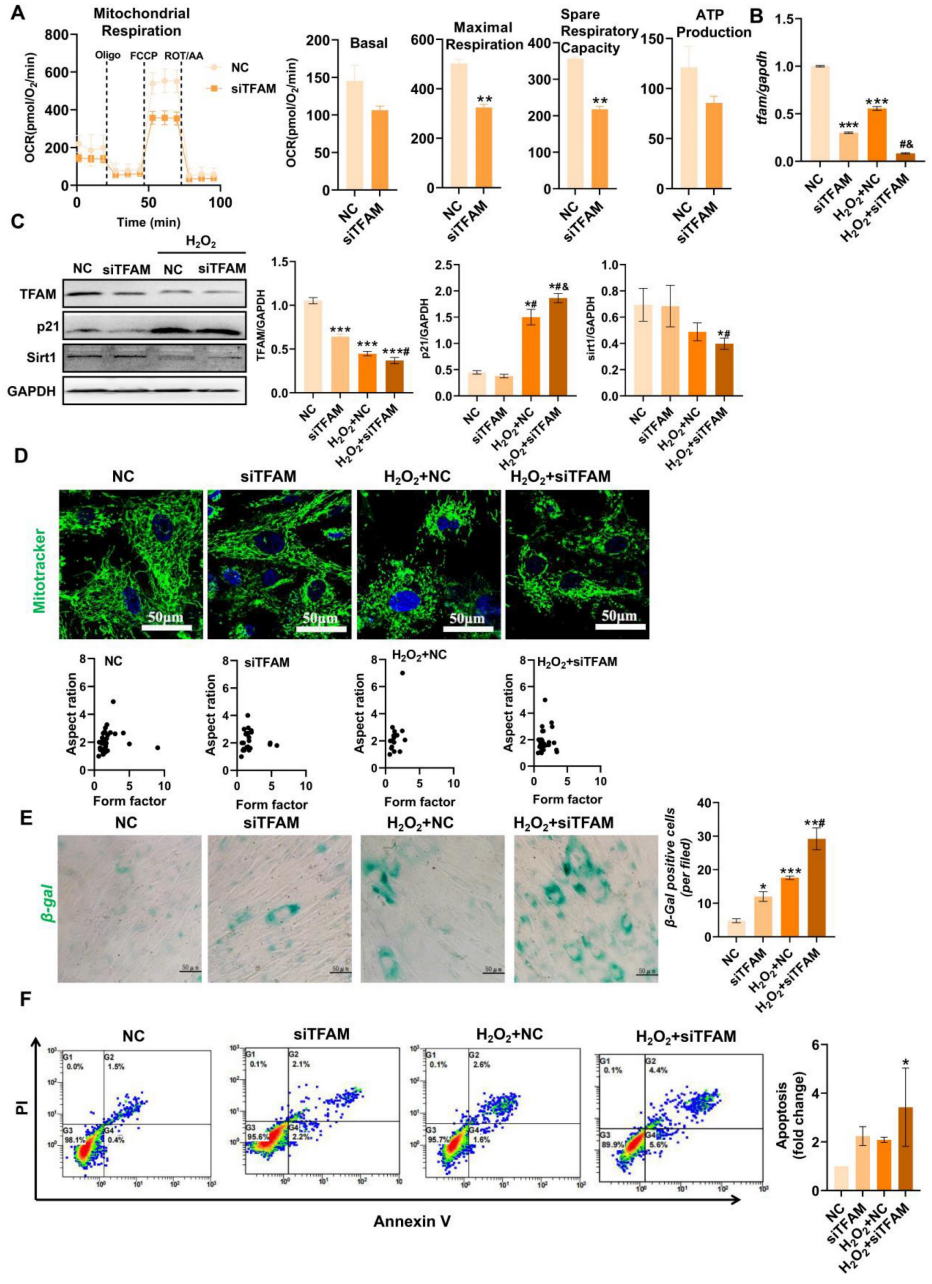
TFAM defects promoted MSC senescence and death under oxidative stress. (A) Measurement of mitochondrial OCR in hMSCs transfected with siTFAM (n = 3; **p < 0.01 *vs.* NC group). (B) Real-time PCR analysis of* TFAM* mRNA in hMSCs treated with siTFAM upon H_2_O_2_ stimulation (n = 3; ***p < 0.001 *vs.* NC group; **^#^**p <0.05 *vs.* siTFAM group; **^&^**p <0.05 *vs.* H_2_O_2_+NC group). (C) Western blot analysis and quantification of the protein levels of the TFAM, p21 and Sirt1 proteins in hMSCs treated with siTFAM upon H_2_O_2_ stimulation (n = 3; *p < 0.05 *vs.* NC group; **^#^**p <0.05 *vs.* siTFAM group; **^&^**p <0.05 *vs.* H_2_O_2_+NC group). (D) Representative micrographs for Mito-Tracker Green staining in hMSCs (scale = 50 µm) and quantification of mitochondrial fragmentation. (E) *β*-gal staining and quantification of hMSCs (scale = 50 µm) (n = 3; *p < 0.05 *vs.* NC group; ***p < 0.001 *vs.* NC group; **^#^**p <0.05 *vs.* siTFAM group). (F) Flow cytometry analysis of apoptotic rates in MSCs (n = 3; *p < 0.05 *vs.* NC group).

TFAM is an essential packaging protein of the mtDNA nucleoid that is necessary for mitochondrial functions, but its loss can disrupt mtDNA homeostasis and result in an overall decline in mitochondrial function 30. Thus, we explored the specific impact of mitochondrial injury on MSC fate using siRNA-mediated TFAM knockdown (siTFAM). Indeed, TFAM defects led to an overall decline in the mitochondrial respiratory function of MSCs even under normal culture conditions (Figure 4A)*.* Importantly, MSCs with TFAM defects were more susceptible to oxidative injury, as indicated by lower levels of mitochondrial biogenesis (as indicated by TFAM and Sirt1 expression) in MSCs of the siTFAM groups than in MSCs of the NC groups under H_2_O_2_ conditions (Figure 4B-C). However, there was no obvious difference in mitochondrial fragmentation between the NC and siTFAM groups under H_2_O_2_ conditions (Figure 4D). This effect might be due to a vicious cycle in which mtDNA injury induces ETC defects and mtROS generation, which in turn promotes mitochondrial damage 12,30. Notably, mitochondrial dysfunction is strongly associated with stem cell senescence and death 9,10, and the levels of TFAM and Sirt1 are markedly reduced during MSC senescence 31,32. Similarly, we found that MSCs in the siTFAM groups had higher levels of p21, *β*-gal^+^ cells and apoptotic rates than those in the NC group under H_2_O_2_ stimulation (Figure 4C, E-F), suggesting that oxidative stress-induced TFAM defects can promote MSC senescence and apoptosis. These results confirm that TFAM defects are vital drivers of MSC loss under oxidative stress, but the underlying mechanism needs to be explored.

### TFAM defects disrupted NEAT1 signaling and paraspeckle formation in MSCs

**Figure 5:**
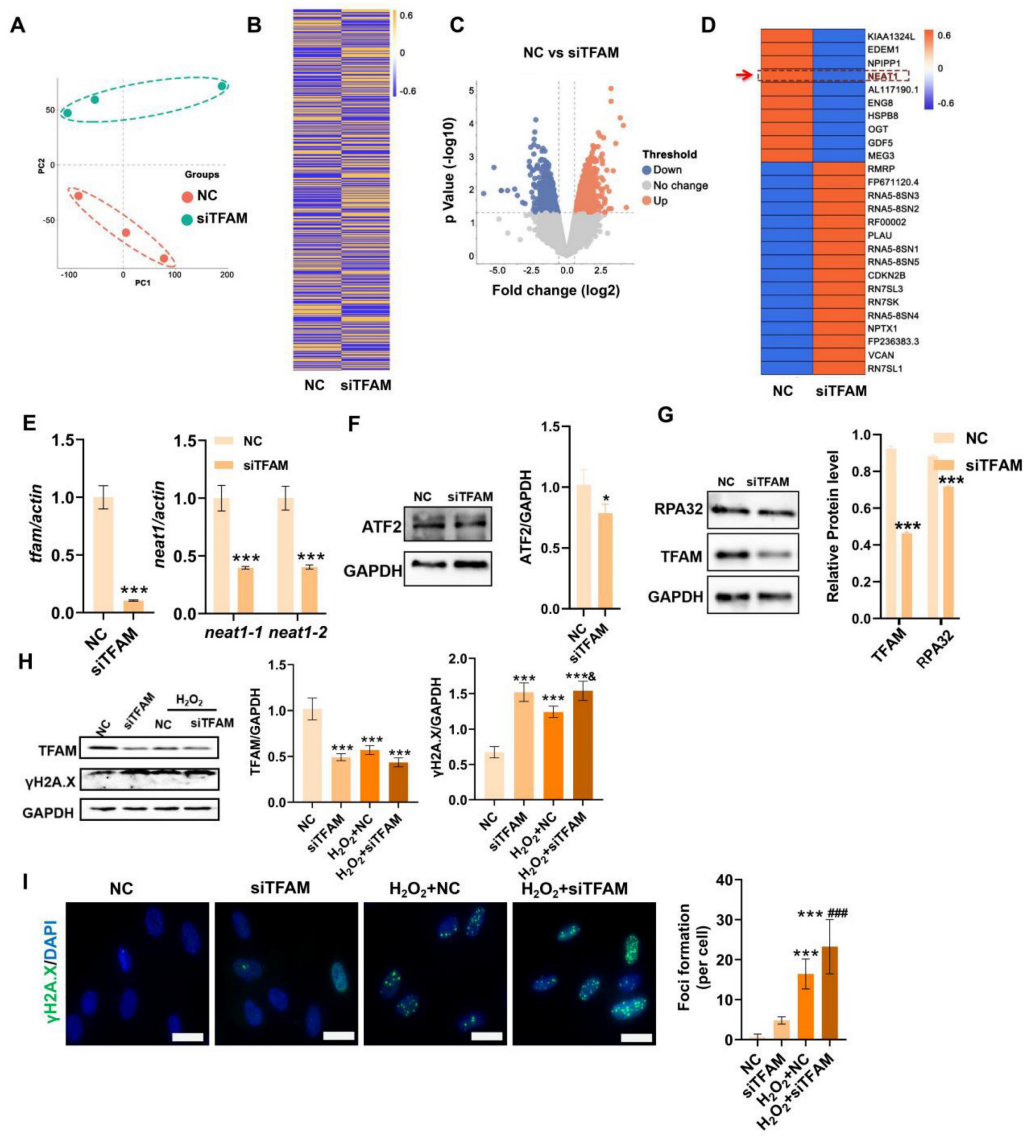
TFAM defects induced NEAT1 depletion and increased DNA damage in MSCs. (A) PCA score plot representing discrepancies between groups (n = 3). (B) Heatmap showing the gene expression pattern of MSCs. (C) Volcano plot identified he differentially expressed genes (DEGs) between groups (fold change >2, p < 0.05). (D) Heatmap showing the top 26 DEGs between groups. (E) Real-time PCR analysis of *TFAM* and *NEAT1* mRNA in hMSCs treated with siTFAM (n = 3; ***p < 0.001 *vs.* NC group). (F-G) Western blot analysis and quantification of the protein levels of the TFAM, ATF2 and RPA32 in hMSCs treated with siTFAM (n = 3; *p < 0.05 *vs.* NC; ***p < 0.001 *vs.* NC group). (H) Western blot analysis and quantification of the levels of the TFAM and γ-H2A.X proteins in hMSCs treated with siTFAM upon H_2_O_2_ stimulation (n = 3; ***p < 0.001 *vs.* NC group; **^&^**p < 0.05 *vs.* H_2_O_2_+NC group). (I) Representative images and quantification of γ-H2A.X levels (scale bar = 50 μm) in hMSCs treated with siTFAM upon H_2_O_2_ stimulation (n = 3; ***p < 0.001 *vs.* NC group; **^###^**p <0.001 *vs.* siTFAM group).

Next, the underlying mechanism that regulates mitochondrial injury-associated MSC death was explored using RNA-seq analysis. The principal component analysis (PCA) plot and heatmap showed clear separation and differential gene expression patterns between the NC group and the siTFAM group (Figure 5A-B). The significantly differentially expressed genes (DEGs) between the NC group and the siTFAM group were further identified (Figure 5C), and the top 26 DEGs are listed in Figure 5D. Interestingly, NEAT1, a critical long noncoding RNA (lncRNA) that controls paraspeckle (PS) formation, was identified as one of the top genes affected by TFAM depletion. Additionally, qPCR confirmed that siTFAM markedly reduced NEAT1 expression in MSCs (Figure 5E). PSs are a type of subnuclear body (~0.2-1 μm in size) that can regulate multiple pathways via sequestration of diverse proteins and RNAs 33. Possible crosstalk between mitochondria and the PS (mitochondria-PS axis) has been found, and depletion of mitochondrial proteins can disrupt NEAT1 expression and PS formation by activating transcription factor 2 (ATF2) pathway 13,34. ATF2 is a nuclear transcription factor that may increase mitochondrial permeability and promote apoptosis under genotoxic stress. For example, knocking down mitochondrial proteins (*e.g.,* ACSM5, ACAD11, GPT2, and MSRB2) decreased the expression of ATF2 and NEAT1 13.

**Figure 6:**
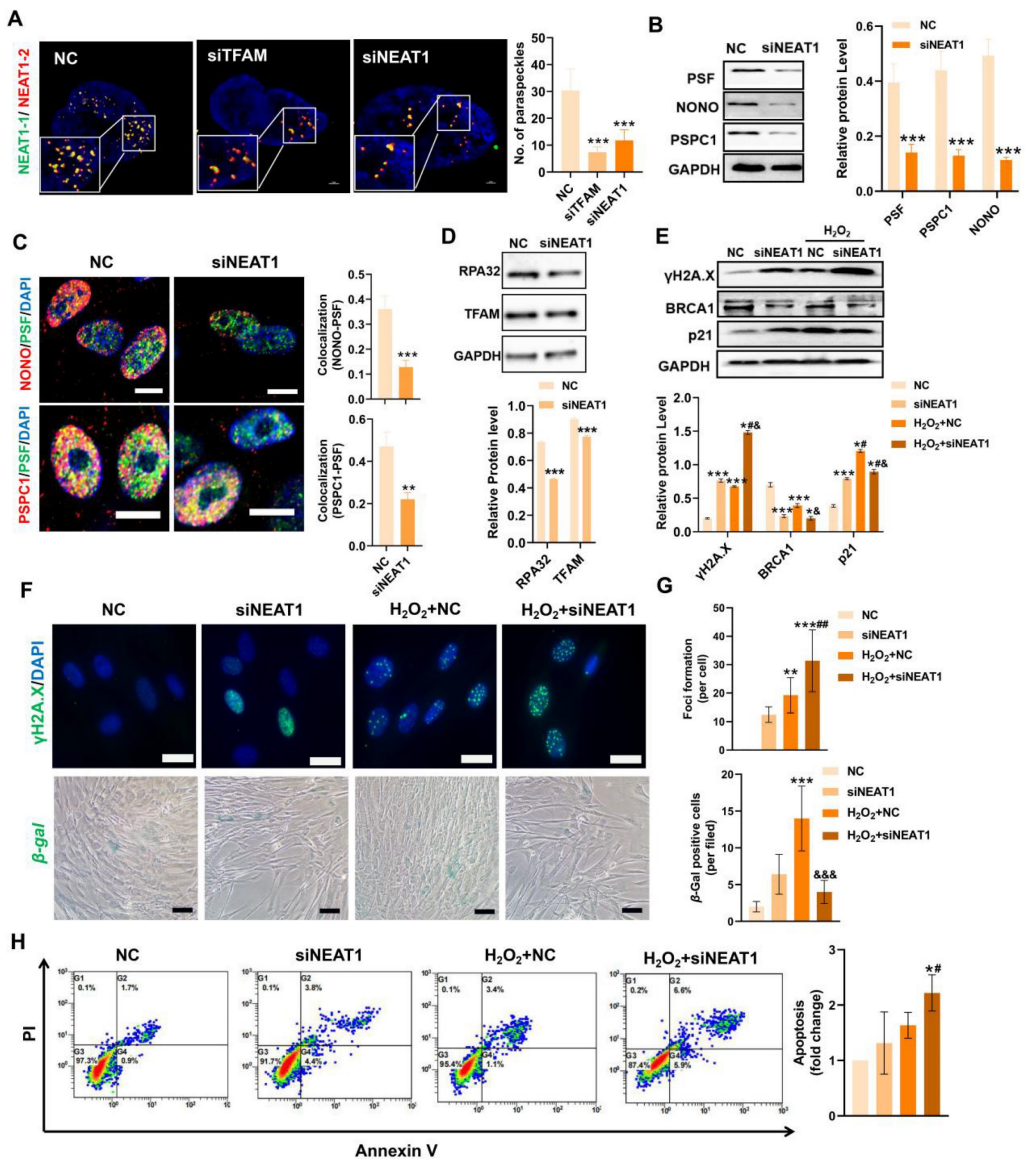
Loss of NEAT1 impaired paraspeckle formation and DNA repair machinery in MSCs. (A) Representative images and quantification of the colocalization of NEAT1-1 and NEAT1-2 by FISH in hMSCs upon siTFAM or siNEAT1 treatment (scale = 2 µm) (n = 3; ***p < 0.001 *vs.* NC group). (B) Western blot analysis and quantification of the levels of the PSF, NONO and PSPC1 proteins in hMSCs treated with siNEAT1 (n = 3; ***p < 0.001 *vs.* NC group). (C) Double-IF staining and colocalization of PSF (green), PSPC1 (red) or PSF (green), NONO (red) in hMSCs treated with siNEAT1 (scale = 10 µm) (n = 3; **p < 0.01 *vs.* NC group; ***p < 0.001 *vs.* NC group). (D) Western blot analysis and quantification of the levels of the RPA32 and TFAM proteins in hMSCs treated with siNEAT1(n = 3; ***p < 0.001 *vs.* NC group). (E)Western blot analysis and quantification of the levels of the γ-H2A.X, BRCA1 and p21 (n = 3; *p < 0.05 *vs.* NC group; ***p < 0.001 *vs.* NC group; **^#^**p <0.05 *vs. s*iNEAT1 group; **^&^**p <0.05 *vs.* H_2_O_2_+NC group). (F) Representative images of γ-H2A.X (scale bar = 50 μm) and *β*-gal staining (scale = 100 µm) of hMSCs treated with siNEAT1 upon H_2_O_2_ stimulation. (G) Quantification of γ-H2A.X and *β*-gal levels in hMSCs (n = 3; ***p < 0.001 *vs.* NC group; **^&&&^**p <0.001 *vs.* H_2_O_2_+NC group). (H) Flow cytometry analysis of MSC apoptotic rates (n = 3; *p < 0.05 *vs.* NC group; **^#^**p <0.05 *vs.* siNEAT1 group).

In contrast, mitochondrial stress induced by FCCP or oligomycin activates ATF2 expression, which subsequently upregulates NEAT1 expression 13. Thus, ATF2 may act as a downstream sensor of mitochondrial signals to modulate NEAT1 expression. TFAM is an essential mitochondrial protein for maintaining mtDNA replication, transcription and mitochondrial function. In this study, we found that knockdown of TFAM led to a reduction in NEAT1 and ATF2 expression (Figure 5E-F), which was consistent with previous findings. Overall, our results suggest that the decrease in NEAT1 following TFAM deletion under ROS conditions is at least partly due to disruption of the mitochondria-ATF2 axis. PSs are subnuclear ribonucleoprotein bodies composed of the lncRNA NEAT1 and core proteins (PSF/SFPQ, NONO, and PSPC1) 35,36. PSs are involved in many cellular processes, such as stress responses (*e.g.,* DNA damage response) and cell apoptosis 33,37. In this study, we found fewer PSs in MSCs from the siTFAM or siNEAT1 groups than in those from the normal groups (Figure 6A). The formation of the PS complex requires a set of additional proteins, such as NONO, PSF and PSPC1, which together with NEAT1 transcripts form a multilayer spheroidal structure 37. It has been reported that depletion of NEAT1 could cause a reduction in paraspeckle numbers, and NEAT1 overexpression increased paraspeckle formation 38. Furthermore, NEAT1 overexpression upregulated NONO and PSF protein expression in cells, meanwhile, the degradation rate of NONO protein was also slower in the presence of translation inhibitor cycloheximide (CHX) 39. These findings indicate that NEAT1 may regulate PS proteins via protein synthesis or degradation pathways. In this study, we found a reduction in the expression of PS proteins (*e.g.,* PSF, NONO and PSPC1) after the knockdown of NEAT1, along with a significant decrease in the number of paraspeckles (Figure 6B-C). These results collectively suggest that NEAT1 might regulate PS protein synthesis and/or degradation pathways, but the detailed mechanism needs to be investigated in future studies. In addition, higher levels of γ-H2A.X (DNA damage sensor) and lower levels of the replication protein A 32 kDa subunit RPA32 (DNA repair protein), compared to those in the NC group, were also found in MSCs from the siTFAM group (Figure 5G-I), suggesting that TFAM defects may disrupt NEAT1-mediated PS formation and the related DNA repair process in MSCs.

The DNA damage response (DDR) is a network of cellular pathways that detect and repair DNA lesions (especially those caused by oxidative stress), and can be activated in response to ROS to protect cells against modest damage. PSs have been proposed to be key regulators of the DNA repair process and apoptosis in response to cell injury 37, while NEAT1 silencing could suppress PS structure formation and cell proliferation and migration in vascular smooth muscle cells 40. It has been reported that depletion of NEAT1 impairs DDR protein (*e.g.,* CHK1, CHK2, RPA32, BRCA1 and RAD51) expression and leads to the accumulation of DNA damage (indicated by the enhancement of γ-H2A.X) in cells 41. Consistent with these findings, we measured increased expression of γ-H2A.X and decreased expression of BRCA1 and RPA32 in MSCs with NEAT1 knockdown (Figure 6D-E). These findings indicate that NEAT1 is responsible for DNA damage and repair. Among the DNA repair types, homologous recombination (HR) is vital one in response to stress and can be affected by NEAT1 signaling 42. For example, knockdown of NEAT1 resulted in a decrease in RPA32 and BRCA1 (a critical mediator of HR repair) expression in cancer cells 37, as well as pRPA32 protein levels 39. These reports indicate that NEAT1 can regulate DNA damage repair, at least partially, via the BRAC1 mediated-HR pathway. Furthermore, PSF (a PS protein that regulates the PS structure) was reported to participate in the HR pathway by promoting the D-loop formation in DNA 39,43. Overall, NEAT1-mediated PS formation could affect the DDR via HR pathway. Altogether, our results suggest that NEAT1-mediated PS formation may regulate the DNA damage repair process by affecting BRCA1/RPA32 expression and their related HR pathway. Under H_2_O_2_ stimulation, MSCs transfected with siNEAT1 had higher levels of DNA damage (γ-H2A.X) and lower levels of DNA repair proteins (*e.g.,* BRCA1) than those in the NC group (Figure 6E-G), suggesting an impairment of the DDR in these cells. It has been reported that overexpression of NEAT1 triggers phosphorylation of RPA32 and CHK2 to promote DNA damage repair through an increase in ATM and DNA-PKcs protein levels. Conversely, loss of NEAT1 impairs DNA repair response 37. To confirm this, activation of NEAT1 was induced by ATF2 overexpression in MSCs (Figure S5A-B), since ATF2 overexpression increased the expression of NEAT1 in BMSCs 44. As a result, ATF2-OE suppressed the expression of p53 protein as well as the TFAM protein in MSCs under normal conditions (Figure S5C). In addition, ATF2-OE partially rescued MSC senescence (Figure S5D-E) but it failed to decrease DNA damage or improve the DDR in MSCs under H_2_O_2_ conditions (Figure S5F). The *in vivo* survival of ATF2-OE MSCs, compared with that of normal MSCs, was shorter (Figure S7). This might be because ATF2 is a nuclear transcription factor that is involved in the regulating diverse cellular processes.

For example, genes (ATM, RAD23B, iNOS, Grp78 and cyclin D1) regulated by ATF2 are involved in stress and DDR 45. Moreover, abnormally high NEAT1 levels may conversely disrupt mitochondrial function and cell survival, since it has been found that excessive NEAT1 signaling has been shown to limit mitochondrial function in young BMSCs 44. Overall, MSCs with DDR defects were more sensitive to oxidative injury, as evidenced by the higher levels of p21, higher number of *β*-gal^+^ cells and higher apoptotic rates in MSCs of the siNEAT1 groups than in the control groups under H_2_O_2_ conditions (Figure 6E-H). Collectively, these results indicate that NEAT1 is critical for maintaining the homeostasis and survival of cells. Thus, NEAT1 expression needs to be tightly regulated (neither too high nor too low) in MSCs. ROS-induced TFAM defects promote MSC loss, at least partially, by impairing NEAT1-mediated PS formation and DDR pathways.

### Restoring TFAM signaling reversed ROS-induced MSC loss to some extent

**Figure 7:**
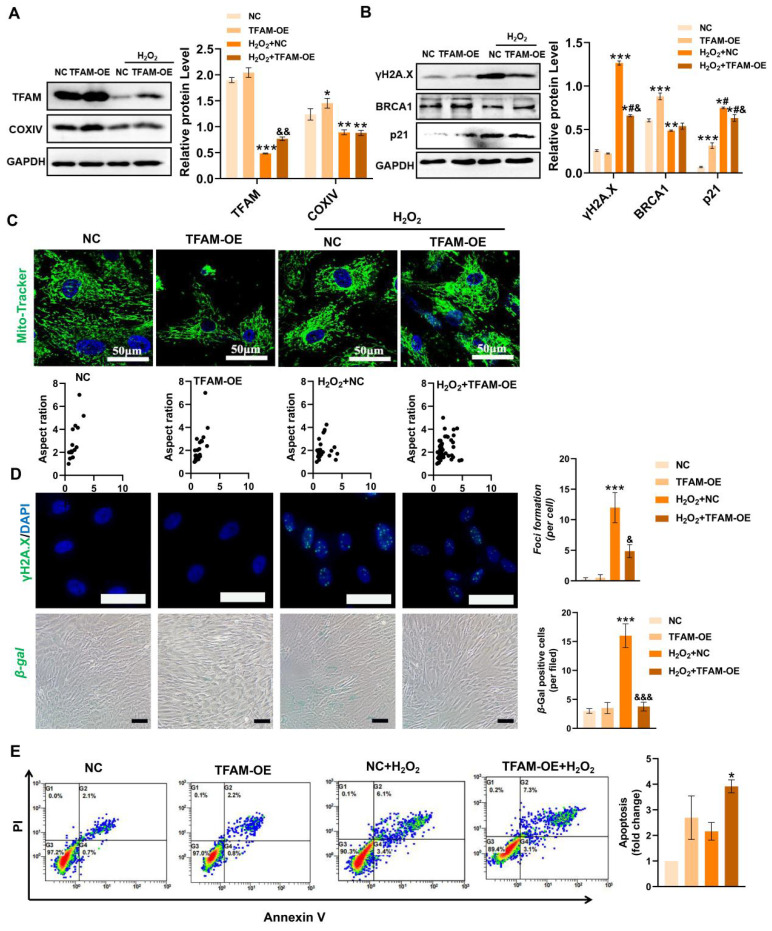
Restoring TFAM reduced ROS-induced MSC senescence and death to some extent. (A) Western blot analysis and quantification of the levels of the TFAM and COXIV proteins in hMSCs transfected with normal control pcDNA (NC) or TFAM-OE plasmid (TFAM-OE) (n = 3; *p < 0.05 *vs.* NC group; **p < 0.01 vs. NC group; ***p < 0.001 *vs.* NC group; **^&&^**p <0.01 *vs.* H_2_O_2_+NC group). (B) Western blot analysis and quantification of the levels of the γ-H2A.X, BRCA1 and p21 proteins in hMSCs (n = 3; *p < 0.05 vs. NC group; **p < 0.01 *vs.* NC group; ***p < 0.001 *vs.* NC group; **^#^**p <0.05 *vs.* TFAM-OE group; **^&^**p <0.05 *vs.* H_2_O_2_+NC group). (C) Representative images of Mito-Tracker Green staining in hMSCs (scale = 50 µm) and quantification of mitochondrial fragmentation. (D) Representative images and quantification of γ-H2A.X (scale bar = 50 μm) and *β*-Gal (scale = 100 µm) levels in hMSCs. (n = 3; ***p < 0.001 *vs.* NC group; **^&^**p < 0.05 *vs.* H_2_O_2_+NC group; **^&&&^**p < 0.001 *vs.* H_2_O_2_+NC group). (E) Flow cytometry analysis of MSC apoptotic rates (n = 3; *p < 0.05 *vs.* NC group).

To confirm the above findings, we also evaluated the effect of TFAM overexpression (TFAM-OE) on DNA damage and MSC loss under oxidative stress conditions. The results showed that TFAM-OE reduced the levels of mitochondrial fragmentation, DNA damage (γ-H2A.X) and cell senescence (p21 and *β*-gal^+^ cells) in MSCs under H_2_O_2_ conditions (Figure 7A-D). Interestingly, TFAM-OE increased COXIV and BRCA1 levels under normal conditions but failed to restore their expression under H_2_O_2_ stimulation (Figure 7A-B). In addition, MSCs with TFAM-OE presented higher NEAT1 levels than normal MSCs (Figure S4A-B), suggesting possible feedback between the TFAM and NEAT1 signaling. These results indicated that TFAM-OE could partially rescue NEAT1-mediated DNA repair processes in MSCs under oxidative stress conditions. However, there was no significant difference in the cell apoptotic rate between the control group and the TFAM-OE group under H_2_O_2_ conditions (Figure 7E). Taken together, our results suggest that TFAM-OE can partly reduce mitochondrial damage and enhance the resistance of MSCs to oxidative injury.

However, genetic modulation of TFAM expression may not be an ideal method for future clinical translation for several reasons. First, the intracellular levels of TFAM need to be fine-tuned since moderate TFAM expression can increase mtDNA copy number and enhance mitochondrial biogenesis 30, while excessive TFAM expression can adversely affect mitochondrial function 46. The transfection efficacy of MSCs, a type of primary cell, is relatively lower than that of other cell lines. Moreover, some reports have raised safety concerns with respect to genetically modified stem cells *in vivo* since virus vector-mediated gene editing may induce immunogenicity and possibly random gene mutations, further increasing the risk of genomic instability and tumor formation in stem cells after transplantation 47,48. Thus, a safer and more efficient strategy that can protect TFAM signaling and the viability of transplanted MSCs needs to be explored.

### Targeted elimination of mtROS improved the survival of transplanted MSCs in vivo

**Figure 8:**
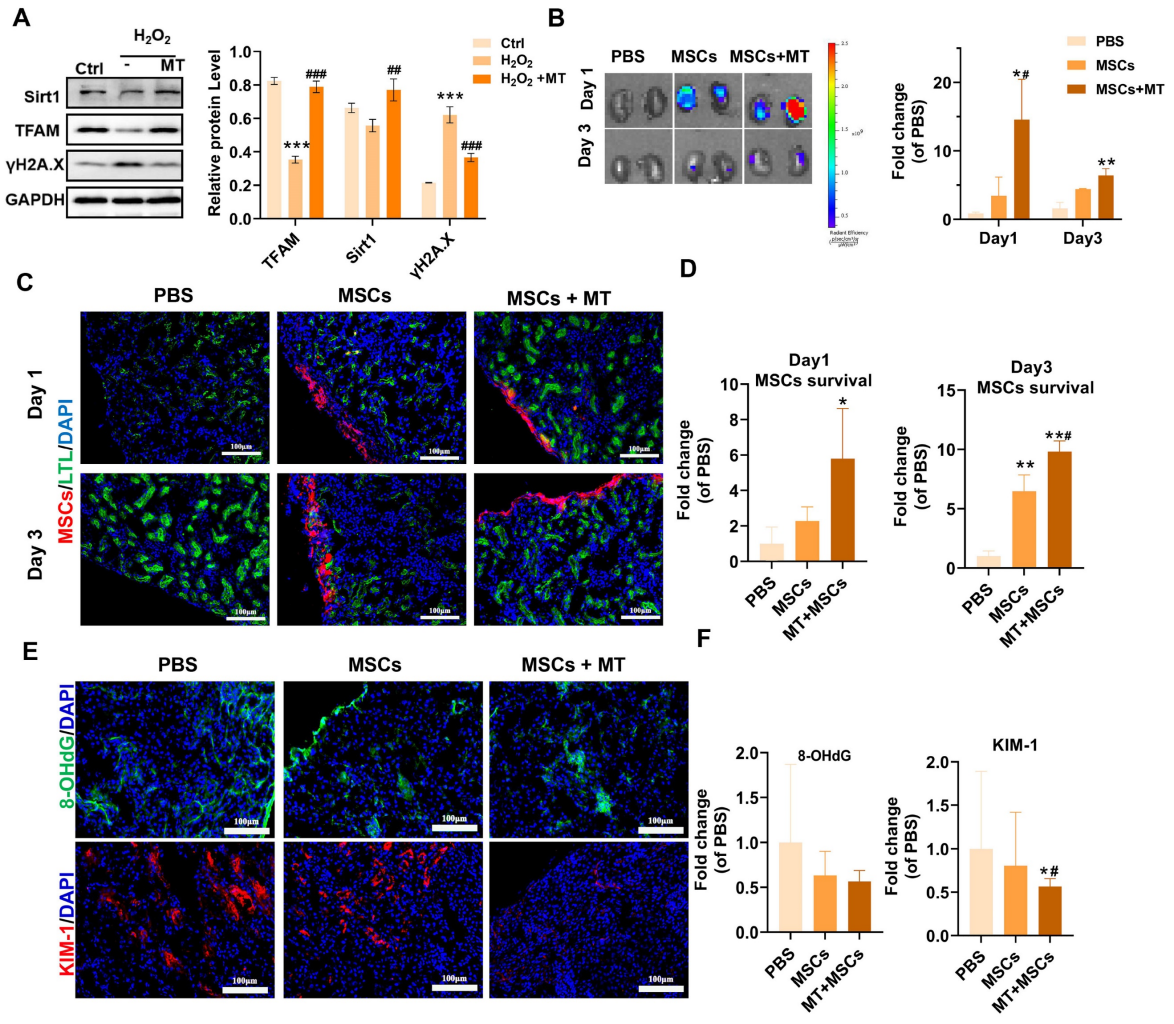
Coadministration of Mito-TEMPO attenuated early transplanted MSC loss. (A) Western blot analysis and quantification of the levels of the Sirt1, TFAM and γ-H2A.X proteins in hMSCs (n= 3; ***p < 0.001 *vs.* Ctrl group; **^##^**p <0.01 *vs.* H_2_O_2_ group; **^###^**p <0.001 *vs.* H_2_O_2_ group). hMSCs were treated with or without MT. (B) Representative IVIS images and quantification of kidneys harvested from mice on Day 1 and Day 3 posttransplantation of DID-labeled MSCs. Mice receiving PBS were included as negative controls (n = 3; *p < 0.05 *vs.* PBS group; **p < 0.01 *vs.* PBS group; **^#^**p <0.05 *vs.* MSCs group). (C-D) Representative micrographs and quantification of MSCs (red) in kidney sections on Day 1 or Day 3. Kidney tubules were stained with LTL (green), and nuclei were stained with DAPI (blue). (E) Representative images of 8-OHdG (green) and KIM-1 (red) IF staining in mouse kidney sections (scale bar = 100 µm). (F) Quantification of 8-OHdG (green) and KIM-1 (red) fluorescence intensity (n = 5; *p < 0.05 *vs.* PBS group; **^#^**p <0.05 *vs.* MSCs group).

Previous studies and our results have shown that implanted MSCs can suffer from oxidative stress in injured tissues and thus exhibit increased mitochondrial dysfunction, DNA damage and senescence. Since the majority of ROS are produced by mitochondria in renal tubular cells, we utilized a mitochondrion-targeted antioxidant (mito-Tempo, MT) to scavenge excessive mtROS in renal tissues following ischemia/reperfusion (I/R). It has been demonstrated that MT treatment can reduce renal mitochondrial oxidative damage in sepsis-induced acute kidney injury and inhibit the expression of p53 in ischemic skeletal muscles 49,50. We previously reported that MT treatment ameliorated mtDNA damage in renal tissues after I/R injury 12. In this study, we also evaluated the effect of MT treatment on DNA damage (indicated by γ-H2A.X) and mitochondrial biogenesis (indicated by sirt1 and TFAM) in MSCs under oxidative stress conditions. We found that MT treatment also restored the expression of TFAM and Sirt1 while reducing DNA damage (γ-H2A.X) levels in MSCs under H_2_O_2_ stimulation (Figure 8A). The results showed that MT treatment improved mitochondrial biogenesis while reducing DNA damage in MSCs under oxidative injury. Next, we sought to determine whether targeted elimination of mtROS can protect transplanted MSCs from oxidative injury *in vivo*. DID-labeled MSCs were injected under the capsule of the ischemic kidneys with or without MT coadministration. As shown in Figure 8B, positive fluorescent signals were detected in the kidneys of mice receiving MSCs, while there were no obvious fluorescent signals in the kidneys of mice receiving PBS (negative control). Interestingly, few fluorescent signals were also found in the lungs, which might be due to the secretomes from the transplanted MSCs (Figure S6). On Day 1 and Day 3 after transplantation, the kidneys of the MSCs + MT group had a higher signal intensity than those of the PBS group or the MSCs alone group. Further histological examination indicated that MSCs were mainly distributed under the renal capsule. From Day 1 to Day 3 after transplantation, the kidneys of the MSC + MT group exhibited higher levels of MSC signals than those of the PBS or MSC alone groups (Figure 8C-D). These results suggest that MT coadministration can reduce the early loss of transplanted MSCs in ischemic kidneys.

The direct antioxidant role of MT in ischemic kidneys was also evaluated. Notably, organ ischemic injury can disrupt mitochondrial ETC function and induce a mtROS burst, which in turn causes severe cellular oxidative injury and cell death 51, thereby contributing to the early loss of transplanted MSCs 7. The kidney is a high energy demanding organ, and a burst of mtROS immediately following renal I/R injury has been reported to occur, which elicits an oxidative stress microenvironment in injured tissues 52. As a result, both renal resident cells (*e.g.,* endothelial cells and tubular epithelial cells) and exogenous cells (*e.g.,* transplanted cells) in injured tissues can experience oxidative damage. Therefore, oxidatively injured cells in ischemic kidneys (including MSCs and resident kidney cells) were detected using 8-OHdG, and kidney injury molecule-1 (KIM-1) was used to indicate the injured kidney tubular areas (Figure 8E-F and S7). As shown in Figure 8E, ischemic kidneys from the MSCs + MT group presented lower levels of 8-OHdG (especially at the site of transplantation under the renal capsule) and KIM-1 expression than those from the PBS group or the MSCs alone group on Day 1 posttransplantation, which suggested the improvement in retention of transplanted MSCs at the ischemic sites was due to attenuation of the oxidative stress microenvironment. However, the reduction in renal tubular injury (as indicated by KIM-1 levels) after MSC plus MT treatment might be a joint effect. MSCs were immediately injected into injured tissues after renal I/R, and they also suffered from high levels of ROS under the same conditions, which was supported by increased levels of 8-OHdG in the kidney (Figure S7, yellow arrow) and MSCs (Figure S7, white arrow). Overall, these results suggest that enhanced oxidative stress occurs after renal I/R injury and can cause oxidative damage to both resident renal cells and transplanted MSCs. Nevertheless, these results suggest that targeted inhibition of mtROS is an efficient strategy for reducing early transplanted MSC loss at injured sites, which may provide insights into advanced stem cell therapy for many forms of tissue injury.

## Conclusion

In summary, disruption of the mitochondria‒paraspeckle axis is a vital reason for early MSC loss under oxidative stress conditions. Briefly, mtROS caused TFAM defects and mitochondrial damage, which further impaired NEAT1-mediated paraspeckle formation and DNA repair machinery, jointly promoting MSC senescence and death. In contrast, MT coadministration reduced oxidative injury in transplanted MSCs and the early loss of these MSCs at the ischemic sites. This study identified the critical role of the mitochondria‒paraspeckle axis in regulating stem cell fate and provided a novel strategy for improving the therapeutic efficacy of stem cell therapy.

## Supplementary Material

Supplementary methods, figures and tables.

## Abbreviations

MSC: mesenchymal stem cell
TFAM: mitochondrial transcription A
NEAT1: nuclear paraspeckle assembly transcript 1
mtDNA: mitochondrial DNA
ROS: reactive oxygen species
mtROS: mitochondrial reactive oxygen species
MT: Mito-Tempo
Ctrl: control
NC: negative control
siTFAM: TFAM siRNA
siNEAT1: NEAT1 siRNA
PSs: paraspeckles
8-OHdG: 8-hydroxy-2'-deoxyguanosine
KIM-1: kidney injury molecule-1
TFAM-OE: TFAM overexpression
ATF2-OE: ATF2 overexpression
SDS-PAGE: sodium dodecyl sulfate‒polyacrylamide gel electrophoresis
PBS: phosphate-buffered saline
IF: Immunofluorescence
