# Supplementary material for: The mitochondria‒paraspeckle axis regulates the survival of transplanted stem cells under oxidative stress conditions
Source: Theranostics. 2024 Jan 27;14(4):1517–33. doi: 10.7150/thno.88764 (PMC10879866; doi:10.7150/thno.88764)
Supplement: Supplementary file 1 — Supplementary methods, figures and tables. [file thnov14p1517s1.pdf]

Supporting information

The mitochondria-paraspeckle axis regulates the survial of transplanted stem cells under oxidative stress conditions

Meng Zhao [a,b], Shuyun Liu [a], Yizhuo Wang [a], Ke Lv [a], Peng Lou [a], Pingya Zhou [a], Jiaying Zhu[b], Lan Li [a], Jingqiu Cheng [a], Yanrong Lu [a], Jingping Liu [a]*

[a] Department of General Surgery and NHC Key Laboratory of Transplant Engineering and Immunology, Frontiers Science Center for Disease-related Molecular Network, West China Hospital, Sichuan University, Chengdu 610041, China

[b] Department of Emergency, Guizhou Provincial People's Hospital, Guiyang 550002, China

*Corresponding Author: Jingping Liu

Address: NHC Key Laboratory of Transplant Engineering and Immunology, West China Hospital, Sichuan University, No. 1 Keyuan 4th Road, Gaopeng Ave, Chengdu 610041, China

Tel: +86-28-85164029, Fax: +86-28-85164030

E-mail: liujingping@scu.edu.cn

Supplemental Methods

Targeted mass spectrometer (MS)-based metabolomics

The metabolites in the cell culture medium were extracted by MeOH/H_2O as previously described[1]. In brief, 50 μL of culture medium was mixed with 250 μL methanol (VWR, Seattle, WA, USA), and the mixture was extracted at -20 °C for 20 min. After centrifugation (14,000 rpm for 10 min), the supernatant was collected and dried in a drier (Eppendorf, Fisher Scientific, Pittsburgh, PA). The dried samples were reconstituted in 150 μL of solution (10 mM ammonium acetate in 40% water/60% ACN + 0.2% acetic acid containing 5.13 μM $^{13}C_2$-tyrosine and 22.5 μM $^{13}C_1$-lactate). Targeted MS-based metabolomics was performed on an Agilent 1260 LC (Agilent Technologies, Santa Clara, CA) coupled to an AB Sciex Qtrap 5500 MS (AB Sciex, Toronto, Canada) system. Fifteen microliters of sample solution was injected into the LC–MS/MS and analyzed under positive and negative ion modes. Chromatographic separations were performed by hydrophilic interaction chromatography (HILIC) using a BEH amide column (2.1 × 150 mm, 2.5 μm, Waters, Milford, MA). The column temperature was set to 40 °C. The mobile phase, gradient conditions and MS parameters were set up as described previously[2]. Multiple reaction monitoring (MRM) mode was used to detect metabolites of interest (total 215 metabolites). The changes in metabolites and the related metabolic pathways were analyzed.

Supplemental Figures

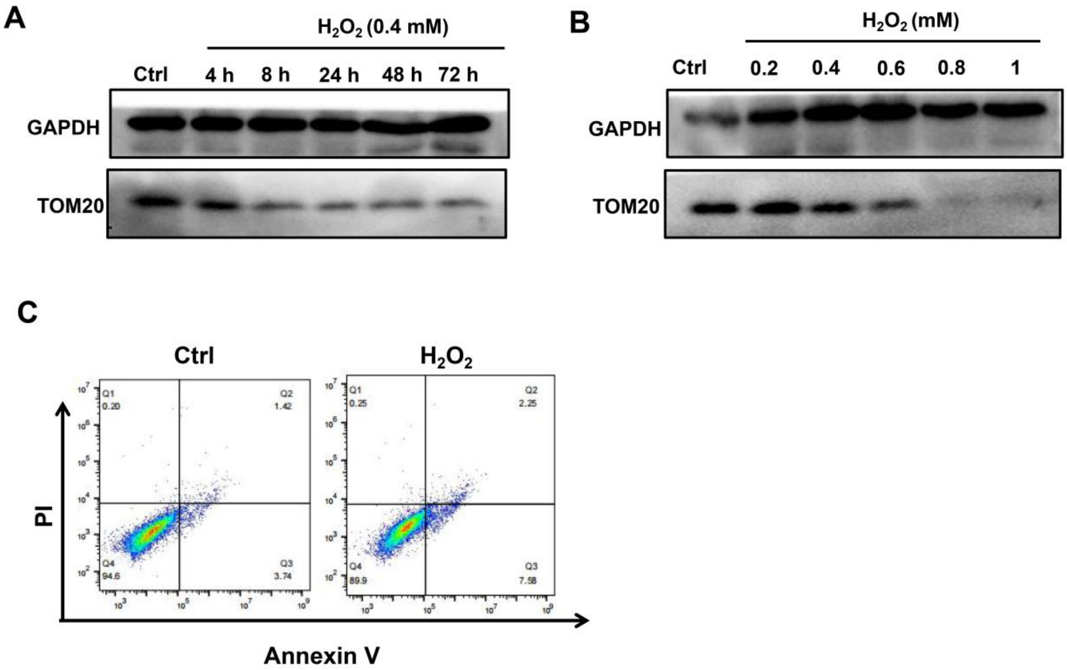

Figure S1. Oxidative stress reduced mitochondrial mass and increased apoptosis in MSCs. (A-B) Western blot analysis of TOM20 protein in MSCs treated with 0.4 mM H_2O_2 for different durations (0 h, 4 h, 8 h, 24 h, 48 h and 72 h) or different H_2O_2 doses (0 mM, 0.2 mM, 0.4 mM, 0.6 mM, 0.8 mM and 1 mM) for 72 h. (C) Flow cytometry analysis of the apoptotic rate in MSCs exposed to 0.4 mM H_2O_2 for 72 h.

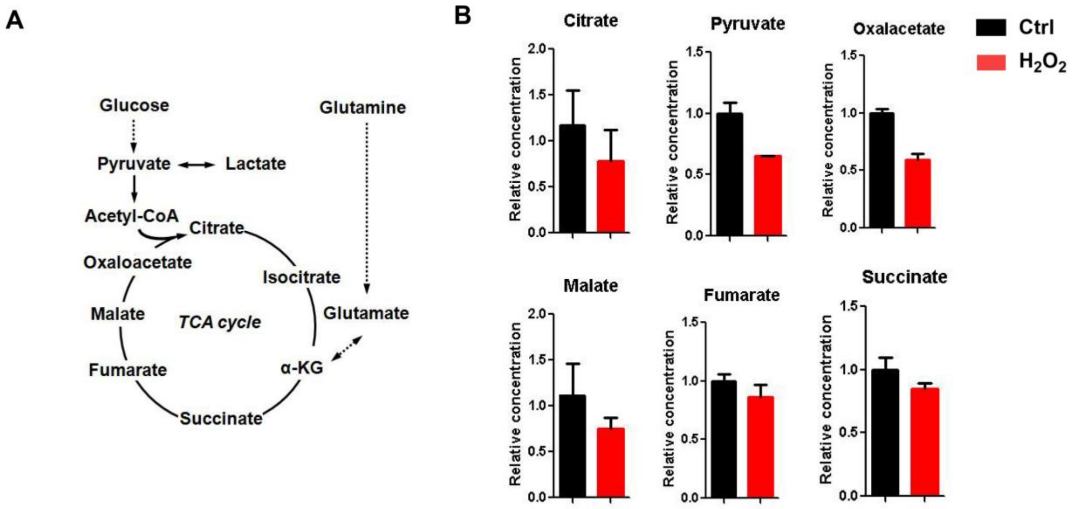

Figure S2. Oxidative stress disrupted TCA cycle metabolite production in MSCs. (A) Schematic diagram of TCA cycle metabolites. (B) LC – MS analysis of citrate, pyruvate, oxalacetate, malate, fumarate and succinate levels in the culture medium of MSCs (n = 4). MSCs were treated with 0.4 mM H_2O_2 for 72 h.

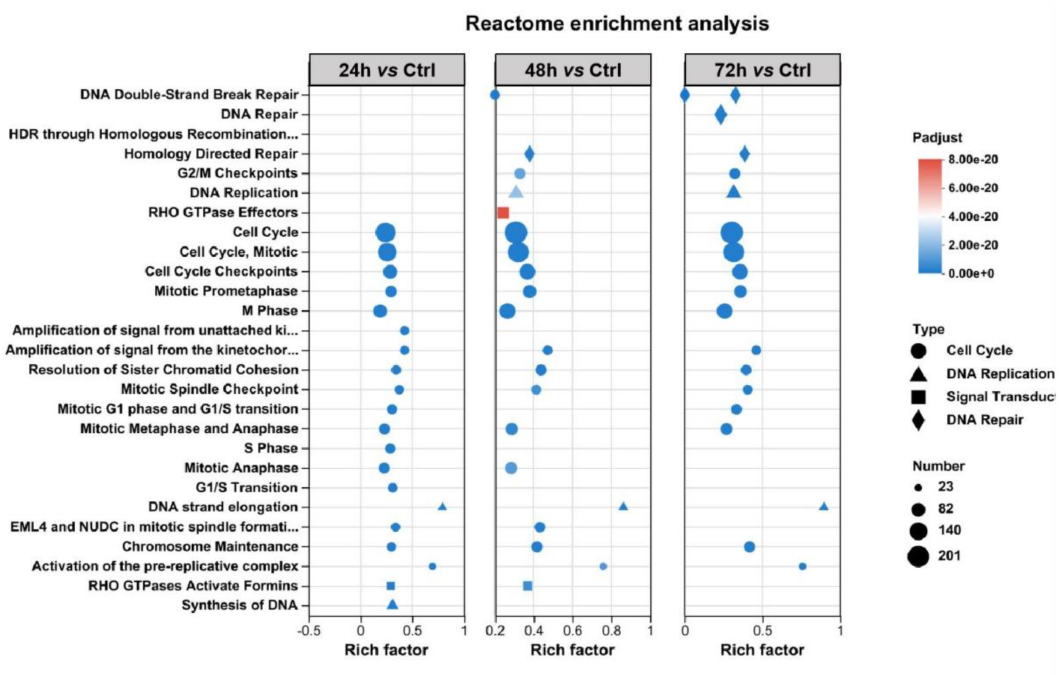

Figure S3. RNA-seq analysis of MSC treated with 0.4 mM H_2O_2 for different durations. Reactome enrichment analysis showing cellular processes and pathwas between two groups.

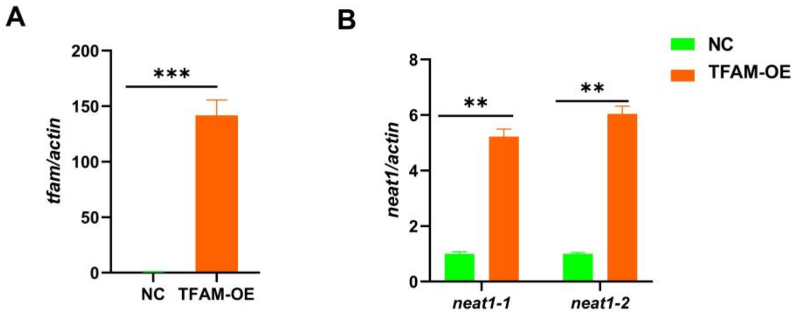

Figure S4. Overexpression of TFAM increased NEAT1 levels in MSCs. MSCs were transfected with negative control plasmid (NC) or TFAM-overexpressing pcDNA plasmid (pcDNA-TFAM). (A) Real-time PCR analysis of TFAM mRNA levels in MSCs (n = 3; ***$p < 0.001$ *vs.* NC). (B) Real-time PCR analysis of NEAT1 levels in MSCs (n = 3; **$p < 0.01$ *vs.* NC).

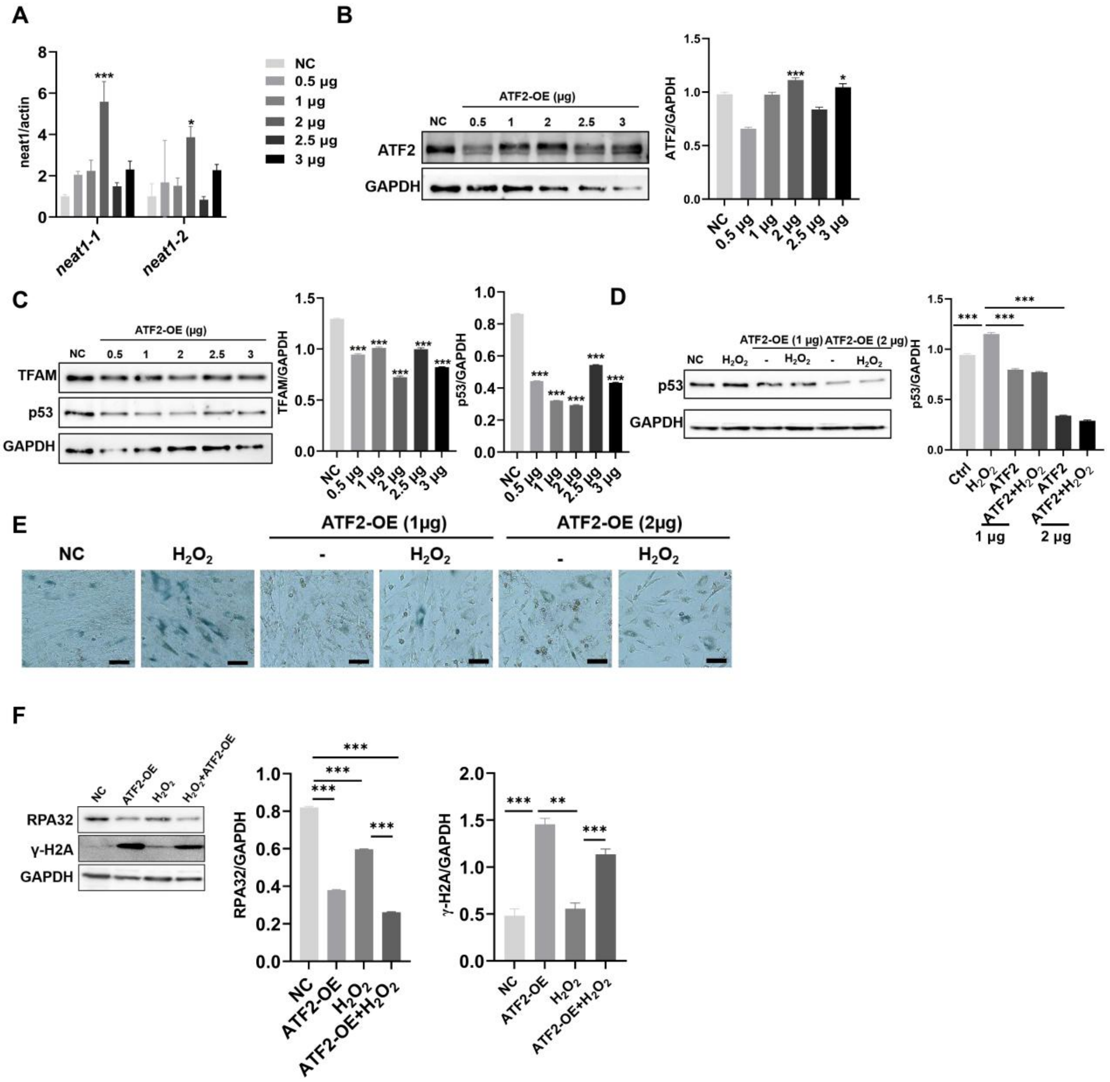

Figure S5. (A) Real-time PCR analysis of the NEAT1 RNA in hMSCs. Cells were transfected with negative control (NC) pcDNA or the ATF2 pcDNA (n = 3, *p < 0.05 *vs.* NC). (B) Western blot analysis of ATF2 protein level in MSCs treated with ATF2 pcDNA. (n = 3,*p < 0.05 *vs.* NC, ***p < 0.001 *vs.* NC). (C) Western blotting analysis of p53 and TFAM protein levels in MSCs treated with ATF2 pcDNA (n = 3, ***p < 0.001 *vs.* NC). (D) Western blotting analysis of p53 protein level in MSCs treated with ATF2 pcDNA plus H_2O_2 (n = 3, ***p < 0.001 *vs.* NC). (E) The expression of β-gal in MSCs was analyzed using a β-gal staining kit (scale bar = 50 μm). (F)

Western blotting analysis of RPA32 and γ-H2A.X protein levels in MSCs treated with ATF2 pcDNA plus H_2O_2 (n = 3, **p < 0.01 *vs.* NC, ***p < 0.001 *vs.* NC)

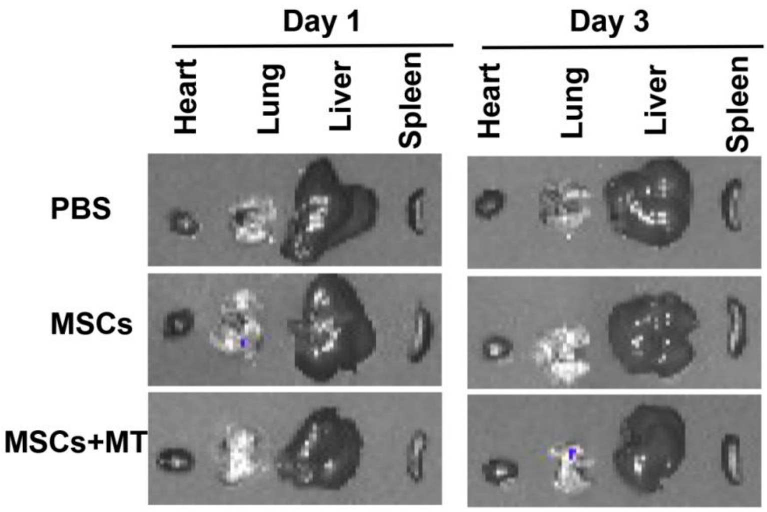

Figure S6. Detection of transplanted MSC signals in major mouse organs. Representative IVIS images of different organs (heart, lung, liver, and spleen) harvested from mice on Day 1 and Day 3 after local injection of DID-labeled MSCs under the injured renal capsule using an insulin syringe. Mice that received PBS alone were used as negative controls.

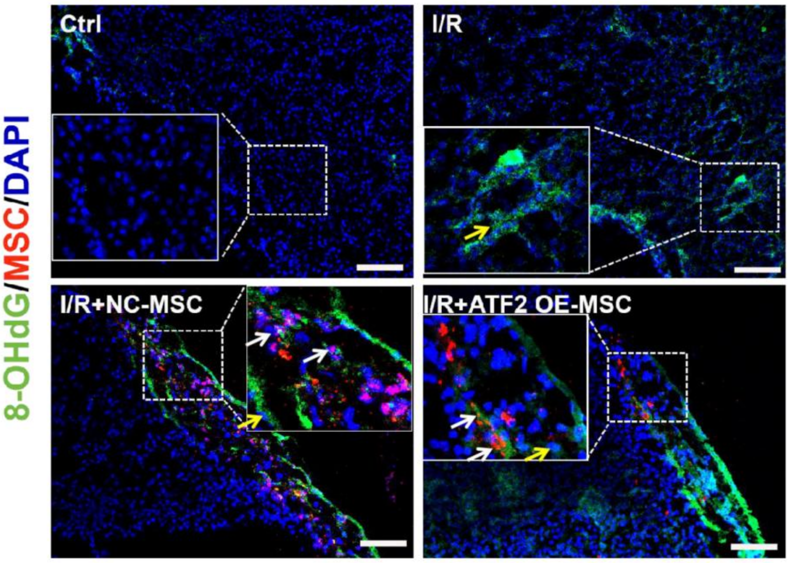

Figure S7. Representative micrographs of 8-OHdG expression in MSCs and renal tissues after I/R injury. DID-labeled MSCs (red) were immediately injected into the renal capsule after renal I/R injury. The colocalization of 8-OHdG (green) and MSCs (red) is indicated (white arrow, scale bar = 100 μm).

Table S1. Real-time PCR primers used in the study

Gene	Sequence 5'-3'	Species
TFAM	AGCTCAGAACCCAGATGCAA	Human
	CCGCCCTATAAGCATCTTGA	
NDUFS8	CATCTACTGCGGCTTCTGC	
	GGGCGTCACCGATACAAGT	
ATP5a-1	AGAGGACAGGAGCCATTGTG	
	TCAGACCAACTCGCCTACG	
NEAT1-1	GGAGGGCCGGGAGGGCTAAT	
	CGGTCAGCCCCGTCGAGCTA	
NEAT1-2	TGACTCTCCATTTCCCCATC	
	TCATTTACCCGCATTTCACA	
ACTIN	GGACTTCGAGCAAGAGATGG	
	AGCACTGTGTTGGCGTACAG	
GAPDH	ACCACAGTCCATGCCATCAC	
	TCCACCACCCTGTTGCTGTA	

Table S2. FISH probes used in the study

Gene	Sequence 5'-3'	Species
NEAT1-1	GACCAGGTAATGTTTTAAGTGA	Human
	AGGCTCAATTTAGAAGATGCAG	
	ACACCTGTGACAAATGAGGAAC	
	TACATGCGTGACTAATACACTC	
NEAT1-2	CAAATGTGTTTGTGAACTCTGC	
	CTGGTATAATAGGTGCTTTTTG	
	GAGAAAGATGCCACTGAATCAC	
	GGATTTGACCAACAAAATGGGG	

References

[1] Liu J, Hanavan P D, Kras K, et al. Loss of SETD2 Induces a Metabolic Switch in Renal Cell Carcinoma Cell Lines toward Enhanced Oxidative Phosphorylation[J]. J Proteome Res, 2019,18(1):331-340.

[2] Du D, Gu H, Djukovic D, et al. Multiplatform Metabolomics Investigation of Antiadipogenic Effects on 3T3-L1 Adipocytes by a Potent Diarylheptanoid[J]. J Proteome Res, 2018, 17(6):2092-2101.
